# Unraveling Cardiovascular Risk in Renal Patients: A New Take on Old Tale

**DOI:** 10.3389/fcell.2019.00314

**Published:** 2019-12-03

**Authors:** Michele Provenzano, Giuseppe Coppolino, Luca De Nicola, Raffaele Serra, Carlo Garofalo, Michele Andreucci, Davide Bolignano

**Affiliations:** ^1^Renal Unit, Department of Health Sciences, University “Magna Graecia” of Catanzaro, Catanzaro, Italy; ^2^Renal Unit, University of Campania “Luigi Vanvitelli”, Naples, Italy; ^3^Interuniversity Center of Phlebolymphology (CIFL), University “Magna Graecia”of Catanzaro, Catanzaro, Italy; ^4^Institute of Clinical Physiology, Italian National Research Council (CNR), Reggio Calabria, Italy

**Keywords:** cardiovascular risk, epidemiology, chronic kidney disease, risk score, smoking habit, statins, eGFR, proteinuria

## Abstract

Chronic kidney disease (CKD), defined by an estimated glomerular filtration rate <60 ml/min/1.73 m^2^ and/or an increase in urine protein excretion (i.e., albuminuria), is an important public health problem. Prevalence and incidence of CKD have risen by 87 and 89%, worldwide, over the last three decades. The onset of either albuminuria and eGFR reduction has found to predict higher cardiovascular (CV) risk, being this association strong, independent from traditional CV risk factors and reproducible across different setting of patients. Indeed, this relationship is present not only in high risk cohorts of CKD patients under regular nephrology care and in those with hypertension or type 2 diabetes, but also in general, otherwise healthy population. As underlying mechanisms of damage, it has hypothesized and partially proved that eGFR reduction and albuminuria can directly promote endothelial dysfunction, accelerate atherosclerosis and the deleterious effects of hypertension. Moreover, the predictive accuracy of risk prediction models was consistently improved when eGFR and albuminuria have been added to the traditional CV risk factors (i.e., Framingham risk score). These important findings led to consider CKD as an equivalent CV risk. Although it is hard to accept this definition in absence of additional reports from scientific Literature, a great effort has been done to reduce the CV risk in CKD patients. A large number of clinical trials have tested the effect of drugs on CV risk reduction. The targets used in these trials were different, including blood pressure, lipids, albuminuria, inflammation, and glucose. All these trials have determined an overall better control of CV risk, performed by clinicians. However, a non-negligible residual risk is still present and has been attributed to: (1) missed response to study treatment in a consistent portion of patients, (2) role of many CV risk factors in CKD patients not yet completely investigated. These combined observations provide a strong argument that kidney measures should be regularly included in individual prediction models for improving CV risk stratification. Further studies are needed to identify high risk patients and novel therapeutic targets to improve CV protection in CKD patients.

## Introduction

Chronic kidney disease (CKD) is defined as the presence of kidney damage, mainly albuminuria, and/or decreased kidney function (estimated glomerular filtration rate [eGFR] <60 mL/min/1.73 m^2^) for at least 3 months (Levey and Coresh, 2012). Since the new onset of eGFR reduction and/or albuminuria is often asymptomatic, CKD is considered as part of non-communicable diseases, together with type 2 diabetes, hypertension and obesity (Jha et al., 2013). In the past decades, the epidemiology of communicable and non-communicable diseases has completely changed. From one side, the drop of mortality due to the main communicable diseases represented the “public health triumph” of the 20th century (Mensah et al., 2017). Indeed, by 1960, the improvement of Health System organization and the development of vaccines and new antibiotics have dramatically decreased mortality from infectious disease and raised life expectancy by an average of 20 years in the United States population (Murray and Lopez, 2013). From around the same period, a sharp decline in cardiovascular (CV) mortality has also been observed in the United States population and worldwide (Centers for Disease Control and Prevention, 1999). In United States, using the 1940 population as reference, the annual heart disease mortality rate declined by 56% in 1996, whereas the mortality rate for stroke fell by 70% in the same period. Similar findings were observed in many other populations (Lozano et al., 2012). The descending trend in CV mortality has been attributed to the improvement in specific prevention strategies, such as better blood pressure control, reduction in total cholesterol plasma levels, smoking cessation, increased physical activity (Ford et al., 2007). On the other side, the burden of non-communicable diseases has dramatically increased in the last quarter century. The number of adults with diabetes has raised from 108 million in 1980 to 422 million in 2014 and that of hypertensive subjects almost duplicated in the period 1975–2015 (NCD Risk Factor Collaboration [NCD-RisC], 2016, 2017). The increased prevalence of these comorbidities together with the population growth and aging, as the longer survival in CV disease, have shaped the CKD epidemiology trend. Indeed, the global prevalence and incidence of CKD has risen by 87 and 89%, respectively, over the period 1990–2016 (Xie et al., 2018). Intriguingly, although the age-standardized mortality rate has shown to be improved for most communicable and non-communicable disease (Ford et al., 2007; Lozano et al., 2012; Mortality and Causes of Death Collaborators, 2015), CKD is among a small number of diseases for which death rates have essentially risen in the last three decades. Specifically, age-standardized death rate due to CKD has increased by 4.39% from 1990 to 2017 (Xie et al., 2018). To date, it has been not completely clarified the specific role of clinical and demographic variables as risk factors for CKD development, maintenance and progression, as well as their effects on the three main endpoints measured in CKD patients: kidney function decline, mortality, CV events. This is, for example, the case of body mass index (BMI) and hypertension. Two recent meta-analyses showed that obesity and not only hypertension, but also pre-hypertension, were significant predictors of new onset CKD (Garofalo et al., 2016, 2017). Further analyses showed that high BMI increases also the risk for dialysis initiation but not for CV events (Russo et al., 2014). These important findings confirm that epidemiologic analyses may provide different results, given the different populations examined.

Aim of this Narrative Review article is to elucidate the main epidemiologic evidences about the association between Kidney damage and CV disease, the mechanisms responsible for this association, available strategies to reduce CV risk in patients suffering from CKD and future directions to answer the unmet needs around this topic.

## Cardiovascular Outcomes in Chronic Kidney Disease Patients

Cardiovascular events remain responsible for a large proportion of unfavorable outcomes in CKD patients. Indeed, among several CKD cohorts, mortality overcomes End-Stage-Kidney-Disease (ESKD), which is considered the “natural” endpoint in these population (Go et al., 2004). Among patients with CKD alone or with CKD and type 2 diabetes of the United States Medicare population, risks of death and atherosclerotic vascular disease exceeded that of ESKD (Foley et al., 2005). Similar findings were reported from individuals with eGFR <60 ml/min/1.73 m^2^ selected from insurance-based health organizations (i.e., Kaiser Permanente Northwest), or Veterans Affair elderly with proteinuria or type 2 diabetes (Keith et al., 2004; Patel et al., 2005). However, these cohorts included patients with advanced age. In the Medicare population, prevalence of patients older than 70, in the subset with CKD and diabetes or CKD alone, was 88 and 91%, respectively (Foley et al., 2005). Cohorts which enrolled patients with a broad range of age, primary renal diseases as well as cohorts of patients referred to Nephrologists (the so-called Nephrology care), showed different results. In a cohort of referred CKD, the rate of ESKD overcame death (including CV death) up to 60 years age or 65 years (only if eGFR was <30 ml/min/1.73 m^2^), being the risk of death more frequent in patients older than 60 years with mild reduced kidney function. Thus, age and kidney function are the two main moderators of mortality and ESKD risks in CKD patients. Moreover, in patients under nephrology care, at variance with unreferred, the ESKD incidence rate is higher than mortality and CV rates (Coppolino et al., 2010; De Nicola et al., 2012, 2017). Nevertheless, CV risk remains relevant in this setting of patients in clinical studies that have been carried-out from different Countries. The presence of previous CV events, defined as myocardial infarction, stroke, heart failure, peripheral vascular disease or revascularization, ranged from 10 to 30% in the NephroTest (France), Mild-to-Moderate Kidney Disease (MMKD) study (Germany, Austria, and South Tyrol) and the British Columbia Study (Canada) (Levin et al., 2008; Dieplinger et al., 2009; Moranne et al., 2009). This prevalence rose to 30–50% in CKD populations of MASTERPLAN (Netherlands) (van Zuilen et al., 2006), CRIB (Chronic Renal Impairment in Birmingham, United Kingdom) (Landray et al., 2001), AASK (African Americans) Study (Norris et al., 2006) and CKD-Multicohort (Italy) (Minutolo et al., 2018); in this latter study, which enrolled CKD patients referred to 40 Italian nephrology clinics, ESKD, CV fatal/non-fatal events and mortality rates were 5.26, 4.52, and 3.76 per 100/pt/years, respectively (Minutolo et al., 2018).

**FIGURE 1:**
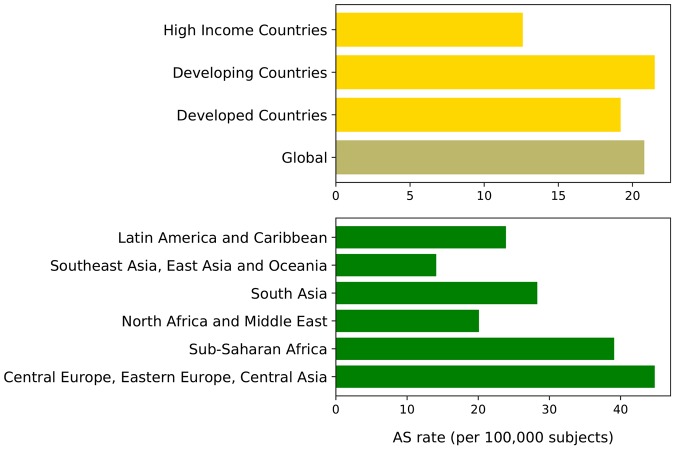
Age-standardized (AS) cardiovascular diseases mortality rate (per 100,000 subjects) worldwide in CKD patients in 2013 (Thomas et al., 2017). Top plot shows rates by development status; bottom plot shows rates by geographic macro-areas. Global, which represents the average rate, is darkly colored. Developed countries are defined as sovereign states that have a developed economy and technologically advanced infrastructure when compared to other countries. (Definition and list at: http://worldpopulationreview.com/countries/developed-countries/). Developing countries are characterized by being less developed industrially with a lower Human Development Index when compared to other countries. (Definition and list at: http://worldpopulationreview.com/countries/developing-countries/). High-income refers to Asia Pacific, Australia, Western Europe, Southern Latin America, North America; sub-Saharan Africa refers to Central sub-Saharan Africa, Eastern sub-Saharan Africa, Southern sub-Saharan Africa, Western sub-Saharan Africa.

Although all these studies evidenced a significant association between CV disease and CKD, the frequency of CV risk varies due to the different inclusion criteria used to recruit patients, particularly with regards of eGFR and proteinuria levels (Astor et al., 2011). An extended estimate of the prevalence of CV risk in CKD patients has been recently realized by the Global Burden of Disease Investigators (Thomas et al., 2017). By analyzing more than 1,000 surveys of CKD patients from around the world, it was found that, in the year 2013, 1,207,453 out 2,163,699 deaths (more than half, 55.8%) were secondary to CV disease. Interestingly, the age-standardized CV disease mortality rate was slightly higher in developing vs. developed world (Figure 1). This phenomenon is partly related to the increase, in developing Countries, of risk factors that can accelerate CV disease development, such as the aging of the population and the change in dietary habit toward high caloric and fatty foods compared to still limited prevention and treatment strategies (Barquera et al., 2015). All these evidences confirm the burden of CV disease in CKD patients and reinforce the need to improve and better understanding how to reduce CV risk in these frailty patients (Piccoli et al., 2018) as well as to ameliorate health care access and infrastructure in developing countries (Perico et al., 2005). Moreover, these findings also encourage incorporating CV disease in the evaluation of the benefits of CKD screening worldwide. Indeed, cost-effectiveness CKD screening studies have concluded that screening is cost effective whether CV fatal and non-fatal events have been included in the analysis at variance with studies that measured the effect on ESKD only (Vos et al., 2010; Crews et al., 2011).

## Chronic Kidney Disease Is an Independent Risk Factor for Cardiovascular Events

In the past decades, several studies affirmed the importance of CKD as a risk factor for the onset of CV events such as myocardial infarction, stroke, chronic heart failure, peripheral vascular disease, CV death. These studies have been carried out in general population and high-risk cohorts (for example diabetic patients, CKD patients and those with a previous CV disease). In a recent meta-analysis, which enrolled more than 100,000 individuals from the general population, both a reduction in kidney function (eGFR) and the presence of kidney damage (albuminuria) were independently and strictly associated with an increased risk of death and CV death, regardless of many potential confounders such as age, gender, and traditional CV risk factors (Chronic Kidney Disease Prognosis Consortium Matsushita et al., 2010). In particular, mortality risk started to increase for eGFR level ≤60 ml/min/1.73 m^2^ and was two-fold higher at 30–45 ml/min/1.73 m^2^ as compared to normal eGFR levels. Regarding the albuminuria-related CV risk, this doubled by moving from 5 to 100 mg/g of urinary albumin/creatinine ratio, thus demonstrating that even a slight increase in albuminuria can herald patients’ higher CV and mortality risk. Similar findings were obtained from high risk population with type 2 diabetes and hypertension (Fox et al., 2012; Mahmoodi et al., 2012; Simeoni et al., 2016; Coppolino et al., 2017). A large meta-analysis from the CKD-Prognosis Consortium assessed the risk of CV mortality by type 2 diabetes status. CV risk was higher in diabetic vs. non-diabetic patients across the whole range of eGFR and albuminuria. However, Authors reported that interaction between eGFR (or albuminuria) and diabetes for all-cause mortality and CV-mortality endpoints was not significant (Fox et al., 2012). This means that, after establishing a reference point on the eGFR or albuminuria scale, the relative risks of the two health outcomes did not differ in diabetic vs. non-diabetic patients. Similar results were obtained when comparing hypertensive vs. non-hypertensive patients (Mahmoodi et al., 2012). An observational analysis of the ADVANCE (Action in Diabetes and Vascular disease: preterAx and diamicroN-MR Controlled Evaluation) clinical trial provided additional useful evidences on CV risk in type 2 diabetes patients. In that study, where more than 10,000 type 2 diabetic patients aged 55 (or older) were enrolled, higher albuminuria and lower eGFR levels were associated with an increased risk of all CV events (CV death, non-fatal myocardial infarction and non-fatal stroke) (Ninomiya et al., 2009).

According to these previous studies, both the two kidney measures (i.e., albuminuria and eGFR) are both strongly associated to the development of CV events. This association is independent from the presence of hypertension and diabetes, thus confirming the crucial role of CKD *per se*.

## Link Between Kidney Measures and Cardiovascular Damage

A first pioneering hypothesis about how albuminuria may testify high CV risk came from the group of researchers of the Steno Memorial Hospital, in Denmark, and was thus called the *Steno hypothesis* (Deckert et al., 1989). Owing the observation that in diabetic patients with increased albuminuria, this marker was associated to an increased transcapillary escape rate of fibrinogen and increased levels of von Willebrand factor, they suggested that albuminuria might reflect a general endothelial dysfunction and systemic vascular damage. Indeed, the leakage of albumin in the vessel-wall may trigger an inflammatory response, thus accelerating the atherosclerotic process. More recently, multiple experimental and clinical studies elucidated that the presence of albuminuria witnesses abnormalities in endothelial glycocalyx, as well as other endothelial structures (Stehouwer et al., 1992; Coppolino et al., 2009; Perticone et al., 2016). Perticone et al. (2015) have also found a significant inverse relationship between alkaline phosphatase and endothelium-dependent vasodilation, which can be mediated by an increase in fibroblast growth factor-23, an early marker of endothelial dysfunction in CKD patients. Moreover, in patients at increased risk for CKD, such as diabetic or hypertensive patients, the microvascular pressure and flows are increased (Ruggenenti and Remuzzi, 2006). This (also called hemodynamic hypothesis) can contribute to the development of albuminuria and the concurrent vascular damage in other organs, such as the heart and the eyes, with the onset of impaired coronary hemodynamics, left ventricular hypertrophy and retinopathy, respectively (Gavin et al., 1998; Liang et al., 2013; De Nicola et al., 2015a).

The contribution of eGFR to the increased CV risk has not completely understood yet, but has raised at the same time an increasing levels of clinical research attention. Indeed, in a survey conducted in the metropolitan area of Kyushu Island, in Southern Japan, heart tissue obtained from 482 individuals who underwent autopsies was examined. The severity of coronary atherosclerosis correlated with the grade of impairment in their kidney function (Nakano et al., 2010). Moreover, the presence of a significant coronary artery stenosis has been found, by angiography, in about half of pre-dialysis patients with extremely low levels of eGFR (Ohtake et al., 2005). Improving management of atherosclerotic risk factors, before reaching an advanced CKD stage, is therefore becoming one of the main targets of Nephrology care.

## CKD as a Risk Equivalent of Cardiovascular Events

A common way to measure a patient’s risk of developing a CV event consists in calculating a 10-year risk based on a combination of some predictors. The Framingham risk score computes the 10-year risk (%) of coronary heart disease for a subject given the exact value of age, gender, systolic blood pressure, total and HDL cholesterol, and smoking status. Once the score has been computed, it should modify the clinical management in accordance to the 10-year CV risk: ≥ 10% defines a very-high risk of CV risk; 5–10% a high risk; 1–5% a moderate risk; < 1% a low risk. However, when the predictive value of the traditional scores have been tested in CKD patients, the risk estimation was suboptimal and, definitely, not well calibrated (Weiner et al., 2007). This means that, traditional CV risk factors are able to explain only a portion of the total CV risk in CKD patients. Therefore, research has recently focused on assessing whether adding the CKD measures (i.e., albuminuria and eGFR) can ameliorate prediction models. A recent meta-analysis of general and high-risk populations, has showed that adding eGFR, albumin-to creatinine ratio (ACR), or both, to the traditional CV risk factors significantly improves the model performance for the prediction of all CV endpoints: CV mortality, coronary heart disease, stroke, and heart failure (Matsushita et al., 2015). More interestingly, the contribution of albuminuria, revealed as ACR, to the overall model performance was greater than the contribution of traditional risk factors. These results are particularly significant and suggest that in population with a higher CV risk, such as CKD patients, the assessment of both eGFR and albuminuria is the essential step. Two additional separate analyses, performed in the general populations of Alberta kidney disease (AKD) and atherosclerosis risk in communities (ARIC), provided important evidences on the relevant contribution of CKD to the development of CV events. Indeed, the AKD showed that the rate of myocardial infarction was significantly higher in patients with CKD, but without diabetes (6.9 per 1,000 person-years), than in those with diabetes, but without CKD (5.4 per 1,000 person-years), being the results similar when the GFR, estimated by the MDRD equation, and sex-specific serum creatinine cutoff points were used to identify reduced GFR (Tonelli et al., 2012). Moreover, the ARIC study evidenced that the adjusted risk for heart failure (HF) was about two-fold higher in patients with eGFR <60 mL/min/1.73 m^2^ compared to those with eGFR ≥90 mL/min/1.73 m^2^ regardless of the presence of coronary heart disease at baseline (Kottgen et al., 2007). Details of the principal observational cohort studies assessing the risk for CV fatal and non-fatal events among CKD patients are shown in Table 1. These results have led guidelines to consider CKD as coronary heart disease risk equivalent. The 2016 ESC/ESH guidelines classified patients with eGFR reduction at high risk (10-year CV mortality risk ≥ 10%), even in absence of albuminuria (Ponikowski et al., 2016). Similarly, KDIGO considered CKD patients older than 50 years old at high risk for CV events (Kidney Disease Improving Global Outcomes Work Group, 2013). Despite the great enthusiasm of such discoveries, several concerns have been raised about considering CKD as a CV disease risk equivalent. Indeed, the CV risk, which was estimated in AKD and ARIC studies, is profoundly influenced by the presence of an established CV disease, that is the strongest cause of a new CV event. Therefore, debate is still ongoing on whether considering CKD as risk equivalent of CV disease or not; in that case more evidence must be obtained to reach that conclusion. We need, in fact, to develop more accurate (i.e., in CKD patients only) risk prediction models on patients’ CV risk and their target treatment, based on these risk scores. New studies in referred CKD patients should comply with this scope.

**TABLE 1:** Observational studies examining rates of cardiovascular (CV) events among CKD patients.

**Study**	**Population**	**Sample size**	**Outcome**	**Results**
Kaiser Permanente Northwest (KPN) (Go et al., 2004)	General population (health care system insuring in Northern California, United States)	1.120.295	Death from any cause, CV events, and hospitalizations	Risks of death, CV events, and hospitalizations increased as the eGFR decreased below 60 ml/min/1.73 m^2^ as compared to eGFR >60 ml/min/1.73 m^2^
United States Medicare population (Foley et al., 2005)	General population (health insurance including 50 United States states)	1.091.201	Incidence of atherosclerotic vascular disease, congestive heart failure or renal replacement therapy	Rates of atherosclerotic vascular disease, congestive heart failure or renal replacement therapy were higher in patients with type 2 diabetes and CKD (or CKD alone) as compared to the groups without CKD. CKD accelerates the progression to all poor outcomes investigated
PREVEND (Brantsma et al., 2008)	General population (Netherlands)	8.496	CV events	Baseline albuminuria and change of albuminuria over time were good predictors of CV events.
Alberta Kidney Disease (Tonelli et al., 2012)	General population (Province of Alberta, Canada)	11.340	Admission to Hospital for myocardial infarction	Rate of incident myocardial infarction in people with diabetes was substantially lower than for those with CKD when defined by eGFR <45 mL/min/1.73 m^2^ and severely increased proteinuria. CKD should be regarded as a coronary heart disease risk equivalent.
HUNT II (Hallan et al., 2007)	General population (Norway)	9.709	CV mortality	Reduced kidney function and microalbuminuria were risk factors for CV death, independent of each other and traditional risk factors.
ARIC (Kottgen et al., 2007)	General population (four US communities)	14.857	Determining the effect of decreased kidney function on HF incidence	The incidence of HF was three-fold higher for individuals with eGFR <60 ml/min/1.73 m^2^ compared to the reference group with eGFR ≥90 ml/min/1.73 m^2^.
Steno (Theilade et al., 2012)	High risk population (Denmark)	900	CV events, mortality, ESRD	Endothelial dysfunction was a predictor of CV events, mortality, ESRD in patients with type I diabetes.
Kaiser Permanente Northwest- CKD (Keith et al., 2004)	CKD patients selected from the KPN cohort	27.998	Renal replacement therapy, death, disenrollment from the health plan	Rate of renal replacement therapy over time was 1.1, 1.3, and 19.9%, respectively, for the CKD stages 2, 3, and 4 whereas the mortality rate was 19.5, 24.3, and 45.7% across CKD stages. Thus, death was far more common than dialysis at all CKD stages.
CKD-Multicohort ^∗^(Minutolo et al., 2018)	Referred CKD patients (40 renal clinics in Italy)	2.174	All-cause mortality, fatal and non-fatal CV events and ESRD	The amount of 24 h proteinuria increased the risk of CV events and ESRD and anticipated the onset of CV events in patients with type II diabetes and CKD.
SIR-SIN (De Nicola et al., 2015b)	Referred CKD patients (100 renal clinic in Italy)	1.306	MACE (CV death, non-fatal events requiring hospitalization; ESRD or 50% eGFR reduction)	eGFR, high LDL cholesterol, type 2 diabetes, old age and previous CV disease predicted CV events in CKD patients.
British Columbia (Levin et al., 2008)	Referred CKD patients (Canada)	4.231	Death, dialysis therapy start, or loss of GFR greater than 5 mL/min/1.73 m^2^/year.	Different clinical or laboratory variables predict kidney disease progression or death in referred CKD patients.

*^∗^ CKD-Multicohort included TArget BLood pressure LEvel in Chronic Kidney Disease (TABLE-CKD) (Minutolo et al., 2006), NEPHROlogy Second University of Naples (NEPHRO-SUN) (De Nicola et al., 2015a), ambulatory blood pressure in chronic kidney disease (ABP-CKD) (Minutolo et al., 2011) and REporting COmorbidities in Renal Disease in ITaly (RECORD-IT) (Minutolo et al., 2013); CV, cardiovascular; CKD, chronic kidney disease; HF, heart failure; ESRD, end stage renal disease.*

## The Tertiary Nephrology Care Setting

**FIGURE 2:**
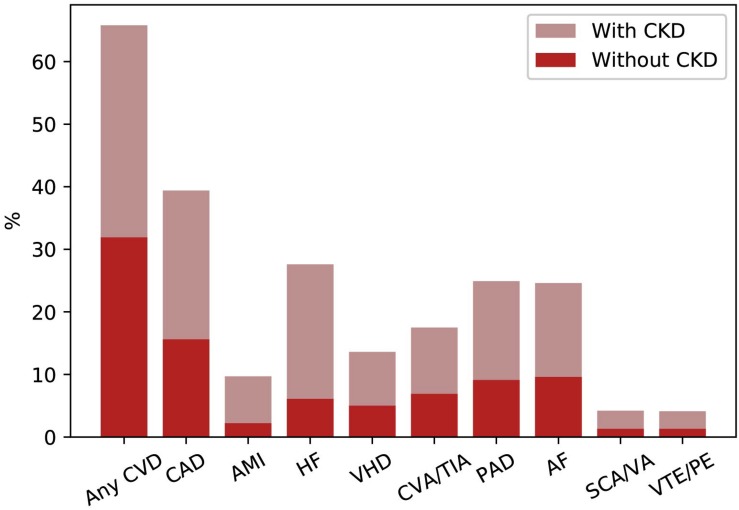
Prevalence of common cardiovascular diseases in patients with or without CKD in United States (2015). AF, atrial fibrillation; AMI, acute myocardial infarction; CAD, coronary artery disease; CKD, chronic kidney disease; CVA/TIA, cerebrovascular accident/transient ischemic attack; CVD, cardiovascular disease; HF, heart failure; PAD, peripheral arterial disease; SCA/VA, sudden cardiac arrest and ventricular arrhythmias; VHD, valvular heart disease; VTE/PE, venous thromboembolism and pulmonary embolism.

**FIGURE 3:**
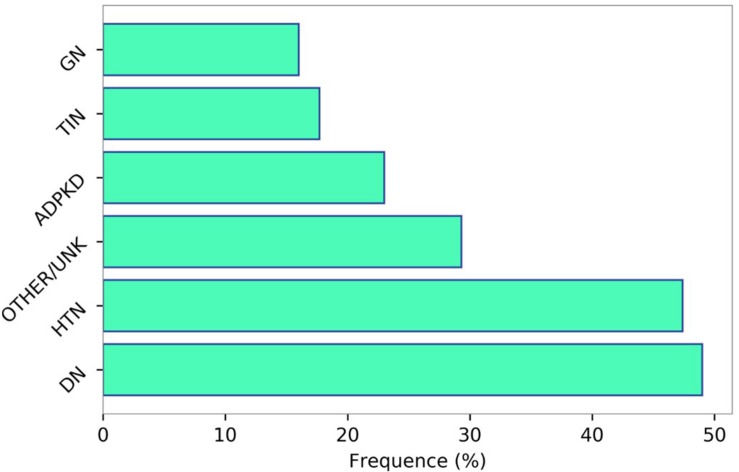
Prevalence of cardiovascular disease (myocardial infarction, stroke, peripheral vascular disease, chronic heart failure, and angina) according to the primary renal disease categories. Data source: 3,957 patients selected from the Italian multicohort of CKD patients referred to nephrologists (Provenzano et al., 2018). HTN, hypertensive nephropathy; DN, diabetic nephropathy; GN, glomerulonephritis; TIN, tubulo-interstitial nephropaties; ADPKD, autosomal dominant polycystic kidney disease; OTHER/UNK, other or unknown diagnosis.

Chronic kidney disease patients under stable “Nephrology care” represent a specific high-risk population that differs from general population and other high-risk populations, such as patients with hypertension or diabetes, in terms of basal risk, management and prognosis. In a multi-cohort analysis of about 4,000 patients referred to Nephrology Units in Italy, we have investigated the frequency of comorbidities and prognosis (Provenzano et al., 2018). Population was characterized by old age on average (67 years), and a high prevalence of diabetes (29%) and CV disease [heart failure, myocardial infarction, peripheral vascular disease, and stroke (34%)]. Therefore, this CKD population, in a large portion of cases, suffers from other comorbidities and consequently is at extremely high risk of worse outcome (Figure 2). A higher CV disease frequency has been found among CKD patients, who were diagnosed on diabetic nephropathy or hypertensive nephropathy, thus demonstrating the presence of a combination of multiple risk factors simultaneously in these patients (Figure 3). On the other hand, among over 400,000 Italian patients, followed by general practitioners, the frequency of diabetes and CV disease was 4.7 and 7.9%, respectively (Minutolo et al., 2008).

Moreover, it has also been shown that in referred CKD patients, CV disease is poorly controlled. In the TABLE-CKD (TArget Blood Pressure Levels), a multicenter study which enrolled patients with stage 3–5 of CKD and at least 6-month follow up of Nephrology care, less than 15% of patients had blood pressure at target (<130/80 mmHg) and more than 80% patients showed an excessive salt intake not counterbalanced by an adequate treatment with loop diuretics, usually prescribed successfully by clinicians (Andreucci et al., 1999). However, the frequency of patients who had a better controlled hypertension significantly increased (up to 75% of patients) after 12 months of Nephrology care, which is generally considered a sufficient time interval for Nephrologists to intervene on the main co-morbidities (Russo et al., 2005; De Nicola et al., 2015a). Similarly, prevalence of hypercholesterolemia reached 50–60% depending on the stage of CKD (De Nicola et al., 2006). In another cohort of patients followed by Nephrologists, the SIR-SIN (Italian study on multiple predictors of outcome- epidemiology of chronic renal insufficiency in Italy) study, the therapeutic inertia for LDL control, defined as the percentage of patients not receiving statin prescription despite being recommended by current guideline, was as high as 61.3% (De Nicola et al., 2015b). These findings confirm, once again, the importance of referring high-risk patients to nephrologists.

**FIGURE 4:**
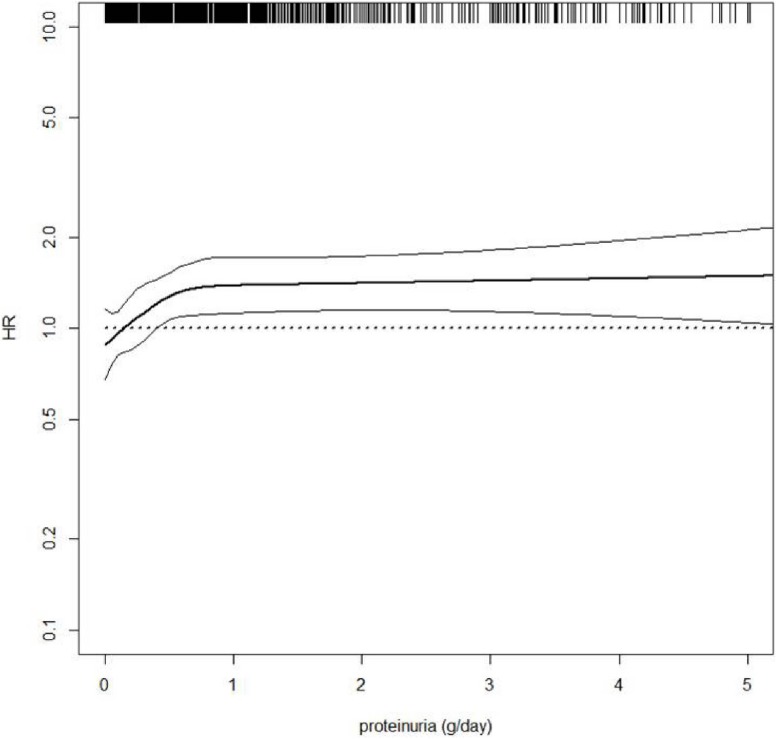
Hazard ratio (HR, solid thick line) and 95% confidence intervals (solid thin lines) for cardiovascular (CV) fatal and non-fatal events (new onset of myocardial infarction, congestive heart failure, stroke, revascularization, peripheral vascular disease, non-traumatic amputation or CV death) by 24 h-proteinuria level. HR was modeled by means of restricted cubic spline (RCS) due to the non-linear association between proteinuria and CV events. Four knots were located at 0, 25th, 50th, and 75th percentiles of proteinuria, whereas HR estimate was adjusted for age, gender, and eGFR value. Rug plot on the *x* axis at the top represents the distribution of observations. Data source: pooled analysis of four cohorts of CKD patients referred to Italian nephrology clinics (Minutolo et al., 2018).

As described for the general population and for diabetic/hypertensive patients, kidney measures have been confirmed as potent predictors of CV outcomes in tertiary nephrology care. The presence of proteinuria is an independent predictor of CV fatal and non-fatal events, with the risk starting to increase from 0.500 g/24 h and from 0.150 g/24 h in non-diabetic-CKD and diabetic-CKD patients, respectively (Coppolino et al., 2017; Minutolo et al., 2018). However, hazard ratio for CV events increased in all patients, selected from this cohort, also for slight increments of 24 h-proteinuria levels (Figure 4). This confirms how the effect of proteinuria on CV outcomes is consistent, strong and independent of other comorbidities, as described in the CKD-Prognosis Consortium. The risk of all-cause and CV death was increased as the eGFR declines (De Nicola et al., 2012). Smoking habit has been considered an independent predictor of CV events in general and high-risk cohorts (Cedillo-Couvert and Ricardo, 2016).

Moreover, current and former smokers have been found at increased risk of developing albuminuria over time as compared to never smokers in the general population (Halimi et al., 2000). However, even though a recent meta-analysis has also shown that smoking is a predictor of new onset of CKD in the general population, the prognostic role of cigarette smoking or the combination of smoking and albuminuria, in patients with already established CKD, remains not completely quantified (Orth and Hallan, 2008; Bolignano et al., 2015; Xia et al., 2017; Coppolino et al., 2018a).

By considering CKD as CV risk equivalent can lead to a reclassification of patients for CV prevention. However, the general opinion, given the differences in basal risks between CKD populations vs. general and high-risk cohorts, is that more studies clarifying the exact role of CV risk factors in referred CKD patients are needed (Nagler et al., 2014). Moreover, despite the fact that to compute a 10-year risk for each patient could be cumbersome, this method can have a greater accuracy in the prediction of CV events in the future, unlike to consider all CKD patients with any degree of proteinuria, eGFR and comorbidities with the same CV risk.

## Strategies for Reducing Cardiovascular Risk in Chronic Kidney Disease Patients

In the last two decades, a growing number of intervention studies have been carried out, with the aim of achieving a better control of CV risk in CKD patients (Mann et al., 2001; Berl et al., 2003; Asselbergs et al., 2004; Norris et al., 2006; Rahman et al., 2006; Solomon et al., 2006; Brugts et al., 2007; Hallan et al., 2007; Kottgen et al., 2007; Perkovic et al., 2007; Bolignano et al., 2008, 2010; Anand et al., 2009; Heerspink et al., 2010; Shiga et al., 2010; Baigent et al., 2011; Parving et al., 2012; Tonelli et al., 2012; Wanner et al., 2016). This point is extremely important because, in the previous CV endpoints-trials, patients with impaired kidney function were regularly excluded (Zoccali et al., 2019). Nowadays, CV risk is also investigated with the aim to find novel biomarkers related to omics, imaging techniques and clinical data, which may help physicians in order to improve their knowledge and management (Serra et al., 2018).

The main intervention studies assessing the CV risk reduction in CKD patients, are listed in Table 2. Interventions differed between studies, with the effect of anti-hypertensive drugs, diuretics, albuminuria lowering agents and statins being tested (Bolignano et al., 2015). The CV benefits of drugs intervening in the renin-angiotensin-aldosterone-system (RAAS-I) are well established. The heart outcomes protection evaluation (HOPE) (Mann et al., 2001)and perindopril protection against recurrent stroke study (PROGRESS) (Perkovic et al., 2007) studies demonstrated, in patients with CKD, that blood pressure lowering using RAAS-I can significantly reduce the subsequent risk for CV events, such as stroke, coronary heart disease, and CV death. Intriguingly, the magnitude of the absolute risk reduction, achieved with that therapy, was greater in patients with CKD than in those without CKD, testifying that the obtained benefit is more pronounced if basal risk is higher. This concept contains a very important “public health” message, namely the priority of identifying CKD patients at increased CV risk that need more intensive management in terms of number of visits and therapy as well (Perkovic et al., 2007). This may also explain why several trials enrolling patients at less increased risk did not show any significant CV protection from the treatment with RAAS-I (Asselbergs et al., 2004; Norris et al., 2006; Bolignano et al., 2016). The most part of these trials have also shown that the risk reduction after blood pressure lowering intervention is strictly dependent on the concomitant albuminuria reduction. An observational analysis of the Reduction in Endpoints in Non-insulin dependent diabetes mellitus with the Angiotensin II Antagonist Losartan (RENAAL) study, a double-blind, randomized clinical trial testing the effect of losartan vs. placebo on cardio-renal outcomes, showed that the change in albuminuria, 6 months after the basal visit, was the strongest determinant of the CV risk reduction (de Zeeuw et al., 2004). Later on, a meta-analysis of 32 RCTs provided a stronger evidence by showing a 13% to 29% risk reduction of CV endpoints for each 10% decrease in albuminuria levels, following different treatments (Savarese et al., 2014). Hence, the CV risk effect of novel antialbuminuric drugs has been tested (Bolignano et al., 2015; Leporini et al., 2016). For this aim, Sodium–glucose cotransporter 2 (SGLT-2) inhibitors are promising agents (Savarese et al., 2014; Wanner et al., 2016; Coppolino et al., 2018b; Garofalo et al., 2019). SGLT-2 inhibitors act by reducing the reabsorption of glucose in the renal proximal tubule (Garofalo et al., 2019). They determine both hemodynamic and non-hemodynamic changes in the kidney, the first being mainly represented by the reduction in intraglomerular pressure and eGFR through tubuloglomerular feedback, whereas the second by a consistent reduction in oxidant stress (up to 60%), a reduction in NLRP3 inflammasome activity and an increase in intrarenal angiotensinogen levels (De Nicola et al., 2014; Bakris, 2019). Large randomized clinical trials assessing the effect of SGLT-2 inhibitors on CV and renal endpoints have shown that they not only can reduce CV risk, but also the progression of renal function decline in patients with CKD and type 2 diabetes. The Canagliflozin and Renal Events in Diabetes with Established Nephropathy Clinical Evaluation (CREDENCE) trial aimed at evaluating the effects of the SGLT2 inhibitor canagliflozin primarily on kidney endpoints in patients with type 2 diabetes and albuminuric CKD. The results of this study have been recently published (Perkovic et al., 2019) and showed that the risk of the primary endpoints (i.e., ESKD, doubling of creatinine, or death from kidney or CV causes) was reduced by 30% with canagliflozin treatment compared to placebo (hazard ratio: 0.70; 95% confidence interval: 0.59–0.82; *p* < 0.001). Findings for the kidney-specific composite endpoint (i.e., ESKD, doubling of creatinine, or kidney-related death) were positive as well (hazard ratio: 0.66; 95% confidence interval: 0.53–0.81; *p* < 0.001). Canagliflozin treatment was also associated with a lower risk for several CV related endpoints, such as the onset of CV death, myocardial infarction, hospitalization for heart failure or stroke (Perkovic et al., 2019).

**TABLE 2:** Intervention studies assessing CV risk reduction in CKD patients.

**Study**	**Population**	**Sample size**	**Intervention**	**Outcome**	**Results**
PROGRESS study (Perkovic et al., 2007)	Cerebrovascular disease and CKD	6.105	Perindopril vs. placebo	Total stroke (fatal or non-fatal) and major vascular events.	Perindopril-based treatment reduced the risk of major vascular events by 30% and stroke by 35% among subjects with CKD, and the absolute effects of treatment were 1.7-fold greater for those with CKD than for those without.
CV outcomes in the irbesartan diabetic nephropathy trial (Berl et al., 2003)	Type 2 diabetic nephropathy and hypertension	1.715	Irbesartan, amlodipine, or placebo.	Doubling of serum creatinine levels, ESRD, and death from any cause.	The composite cardiovascular event rate did not differ in patients with type 2 diabetes and overt nephropathy treated with irbesartan, amlodipine, or placebo in addition to conventional antihypertensive therapy.
ALLHAT study (Rahman et al., 2006)	CKD and hypertension	31.797	Chlorthalidone vs. amlodipine vs. lisinopril.	To compare rates of CHD and ESRD; to determine whether GFR independently predicts risk for CHD; and to report the efficacy of first-step treatment with a CCB or an ARB each compared with a diuretic in modifying CV disease.	Older high-risk patients with hypertension and reduced GFR are more likely to develop CHD than to develop ESRD. A low GFR independently predicts increased risk for CHD. Neither amlodipine nor lisinopril is superior to chlorthalidone in preventing CHD, stroke, or combined CV disease, and chlorthalidone is superior to both for preventing heart failure.
ADVANCE study (Heerspink et al., 2010)	CKD and type 2 diabetes	10.640	Perindopril and indapamide vs. placebo	Major adverse cardiac event or MACE	The treatment benefits of a routine administration of a fixed combination of perindopril–indapamide to patients with type 2 diabetes on cardiovascular and renal outcomes, and death, are consistent across all stages of CKD at baseline. Absolute risk reductions are larger in patients with CKD highlighting the importance of blood pressure-lowering in this population.
SHARP study (Baigent et al., 2011)	CKD	9.270	Simvastatin and ezetimibe vs. placebo	Major adverse cardiac event or MACE	Reduction of LDL cholesterol with simvastatin 20 mg plus ezetimibe 10 mg daily safely reduced the incidence of major atherosclerotic events in a wide range of patients with advanced chronic kidney disease.
EMPA-REG OUTCOME study (Wanner et al., 2016)	Type 2 diabetes at high risk for cardiovascular events	7.020	10 mg Empagliflozin vs. 25 mg of empagliflozin vs. placebo	Incident or worsening nephropathy (progression to macroalbuminuria, doubling of the serum creatinine level, initiation of renal-replacement therapy, or death from renal disease) and incident albuminuria.	In patients with type 2 diabetes at high CV risk, empagliflozin was associated with slower progression of kidney disease and lower rates of clinically relevant renal events than was placebo when added to standard care.
CREDENCE study (Perkovic et al., 2019)	type 2 diabetes and albuminuric CKD	4.401	Canagliflozin 100 mg/day vs. placebo	Composite of end-stage kidney disease (dialysis, transplantation, or a sustained estimated GFR of <15 ml/min/1.73 m2), a doubling of the serum creatinine level, or death from renal or cardiovascular causes.	Relative risk (RR) for the primary outcome was 30% lower in canagliflozin group vs. placebo. Renal specific outcome RR was lower by 34% in canagliflozin group vs. placebo. Canagliflozin also reduced risk for cardiovascular death, myocardial infarction, or stroke and hospitalization for heart failure.
HIJ-CREATE study (Shiga et al., 2010)	High-risk hypertensive patients with CHD and CKD	1.022	Candesartan vs. non-ARB treatment	Major adverse cardiac event or MACE	There was no difference in MACE between the two arms in patients without impaired renal function. However, there was a lower incidence of MACE in the candesartan-based treatment arm than in the non-ARBs treatment arm in patients with impaired renal function.
ALTITUDE study (Parving et al., 2012)	Type 2 diabetes and CKD, CVD, or both	8.561	Aliskiren vs. placebo in addition to an ACE inhibitor or an ARB	Major adverse cardiac event or MACE ESRD, death attributable to kidney failure, or the need for renal-replacement therapy with no dialysis or transplantation available or initiated; or doubling of the baseline serum creatinine level.	The addition of aliskiren to standard therapy with renin-angiotensin system blockade in patients with type 2 diabetes who are at high risk for cardiovascular and renal events is not supported by the study and may even be harmful.
HOPE study (Mann et al., 2001)	CKD and non-CKD patients	9.287	Ramipril vs. vitamin E vs. placebo and vitamin E vs. placebo	Major adverse cardiac event or MACE; effect of ramipril on reducing CV risk.	In patients who had preexisting vascular disease or diabetes combined with an additional cardiovascular risk factor, mild CKD significantly increased the risk for subsequent cardiovascular events. Ramipril reduced CV risk without increasing adverse effects.
EUROPA study (Brugts et al., 2007)	CKD and non-CKD patients with stable CHD	12.056	Perindopril vs. Placebo	Major adverse cardiac event or MACE (cardiovascular death, non-fatal myocardial infarction, unstable angina, heart failure, stroke and other cardiovascular events requiring hospitalization).	Treatment benefits of perindopril were apparent in both patient groups either with eGFR ≥75 or eGFR <75. Has not been observed significant interaction between renal function and treatment benefit. The treatment benefit of perindopril is consistent and not modified by mild to moderate renal insufficiency.
PEACE study (Solomon et al., 2006)	CKD and non-CKD patients with stable CHD	8.290	Trandolapril vs. placebo	Major adverse cardiac event or MACE	Trandolapril was associated with a reduction in total mortality in patients with reduced renal function but not in patients with preserved renal function.
Val-HeFT study (Anand et al., 2009)	CKD and non-CKD patients with HF	5.010	Valsartan vs. placebo	Death and first morbid event, defined as death, sudden death with resuscitation, hospitalization for HF, or administration of intravenous inotropic or vasodilator drugs for 4 h or more.	Valsartan reduced the eGFR by the same amount in patients with and without CKD and reduced the risk of the first morbid event in patients with CKD, which suggests its beneficial effects in patients with HF and CKD.
AASK study (Norris et al., 2006)	African Americans with hypertensive nephrosclerosis.	1.094	Metoprolol vs. ramipril vs. Amlodipine	Major adverse cardiac event or MACE	Neither randomized class of antihypertensive therapy nor BP level had a significant effect on the occurrence of CV events. In multivariable analyses, seven baseline risk factors remained independently associated with increased risk for the CV events: BP level, duration of hypertension, abnormal ECG result, non-HDL cholesterol level, BUN, urine protein-creatinine ratio, urine sodium-potassium ratio, and low annual income.
PREVEND IT study (Asselbergs et al., 2004)	Microalbuminuric subjects	864	Fosinopril vs. placebo	Major adverse cardiac event or MACE	In microalbuminuric subjects, treatment with fosinopril had a significant effect on urinary albumin excretion. In addition, fosinopril treatment was associated with a trend in reducing cardiovascular events.
MASTERPLAN (Peeters et al., 2014)	multifactorial approach with the aid of nurse-practitioners reduces cardiovascular risk in patients with CKD	788	Nurse practitioner support added to physician care vs. physician care alone	Death, ESRD, and 50% increase in serum creatinine	The intervention reduced the incidence of the composite renal endpoint by 20% additional supporting that nurse practitioners may attenuate the decline of kidney function and improved renal outcome in patients with CKD

*MACE defined as: cardiovascular death, non-fatal myocardial infarction, unstable angina, heart failure, stroke, and other cardiovascular events requiring hospitalization; CV, cardiovascular; CKD, chronic kidney disease; HF, heart failure; ESRD, end stage renal disease; CHD, coronary heart disease; CCB, calcium-channel blocker; ARB, angiotensin receptor blockers.*

Interestingly, since these drugs modify pathways (mainly the intraglomerular hypertension) that are shared between diabetic and non-diabetic CKD patients, it has been started the attempt of evaluating their benefits even in CKD patients without diabetes. Two large RCTs are actually ongoing and their results are eagerly expected. The Dapa-CKD clinical trial (ClinicalTrials.gov Identifier: NCT03036150) is testing the effects of the SGLT-2 inhibitor dapagliflozin on kidney endpoints and CV mortality in patients with CKD with and without type 2 diabetes vs. placebo, whereas the EMPA-KIDNEY trial is exploring empagliflozin vs. placebo in patients with CKD in order to answer the question whether SGLT-2 inhibitors are effective in a wide range of patients with CKD, such as patients with albuminuria or with reduced eGFR regardless of albuminuria levels (Herrington et al., 2018).

One of the reasons why the current available treatments for CKD patients can reduce, but not completely delete, the CV risk is due to the wide variability in response to treatments. It has been demonstrated from a pooled analysis of clinical trials that almost 30% of patients do not respond to antialbuminuric or blood pressure lowering drugs (Minutolo et al., 2000; Petrykiv et al., 2017). Statins have demonstrated to reduce CV risk in CKD patients. In the SHARP clinical trial, patients with CKD, who underwent statin treatment, were significantly protected from CV events as compared to placebo group, regardless of the basal level of LDL cholesterol (Baigent et al., 2011). KDIGO guidelines have therefore recommended to start treatment based on the CV risk and eGFR level, rather than on the LDL-cholesterol target. Evidences providing protective LDL targets, in CKD patients, deserve a further research effort.

## Conclusion

In conclusion, knowledge on the association between CKD and CV risk has improved in the last years: (1) CKD is an independent risk factor of CV outcomes; the “public health” dimension of this concept is enormous because prevalence of CKD has dramatically increased (Provenzano et al., 2019); (2) identifying high risk patients is a priority; further observational studies are needed to gain more insights on the role of the main risk factors, specifically assessed in CKD; (3) common CV risk calculators are not useful in CKD patients; since CKD patients manifest different CV risk according to the presence of comorbidities, computing risk calculators specific to CKD is required. This may help in risk stratification and clinical decision making; (4) new clinical trials, aimed to reduce the CV risk excess in CKD patients are ongoing. In this regard, efforts are required to reduce variability in response to the available nephro- and cardio-protective treatments.

## Author Contributions

DB, GC, LD, RS, and CG contributed to the research idea. MP and GC wrote the manuscript. MA and DB critically revised the content of the manuscript.

## Conflict of Interest

The authors declare that the research was conducted in the absence of any commercial or financial relationships that could be construed as a potential conflict of interest. The reviewer GR declared a past co-authorship with several of the authors MP, LD, and CG to the handling Editor.
